# New Insights into Adult and Paediatric Chronic Non-bacterial Osteomyelitis CNO

**DOI:** 10.1007/s11926-020-00928-1

**Published:** 2020-07-23

**Authors:** Christian M. Hedrich, Henner Morbach, Christiane Reiser, Hermann J. Girschick

**Affiliations:** 1 Department of Women’s & Children’s Health, Institute of Translational Medicine, University of Liverpool, Liverpool, UK; 2 Department of Paediatric Rheumatology, Alder Hey Children’s NHS Foundation Trust Hospital, East Prescot Road, Liverpool, L14 5AB Great Britain UK; 3 Pediatric Rheumatology and Immunology, Department of Pediatrics, University of Würzburg, Josef-Schneider-Str.2, 98080 Würzburg, Germany; 4 Department of Pediatrics, Pediatric Rheumatology and Immunology, Landeskrankenhaus Bregenz, Carl-Pedenz-Straße 12, 6900 Bregenz, Austria; 5 Vivantes Klinikum Friedrichshain, Children’s Hospital, Landsberger Allee 49, 10249 Berlin, Germany; 6 University Childrens Hospital, Julius Maximilians Universität Würzburg, Würzburg, Germany

**Keywords:** Chronic non-bacterial osteomyelitis, Chronic recurrent multifocal osteomyelitis, Bone autoinflammation, Lymphoplasmacellular osteomyelitis

## Abstract

**Purpose of Review:**

To describe in detail the clinical synopsis and pathophysiology of chronic non-bacterial osteomyelitis and SAPHO syndrome.

**Recent Findings:**

Chronic non-bacterial osteomyelitis (CNO) has been identified as a disease entity for almost 50 years. This inflammatory bone disorder is characterized by osteolytic as well as hyperostotic/osteosclerotic lesions. It is chronic in nature, but it can present with episodic flairs and phases of remission, which have led to the denomination “chronic recurrent osteomyelitis”, with its severe multifocal form “chronic recurrent multifocal osteomyelitis” (CRMO). For almost three decades, an infectious aetiology had been considered, since especially *Propionibacterium acnes* had been isolated from bone lesions of individual patients. However, this concept has been challenged since long-term antibiotic therapy did not alter the course of disease and modern microbiological techniques (including PCR) failed to confirm bone infection as an underlying cause. Over recent years, a profound dysregulation of cytokine expression profiles has been demonstrated in innate immune cells of CNO patients. A hallmark of monocytes from CNO patients is the failure to produce immune regulatory cytokines interleukin-10 (IL-10) and IL-19, which have been linked with genetic and epigenetic alterations. Subsequently, a significant upregulation of pro-inflammatory, NLRP3 inflammasome-dependent cytokines (IL-1β and TNF-α), has been demonstrated.

**Summary:**

The current knowledge on CNO, the underlying molecular pathophysiology, and modern imaging strategies are summarized; differential diagnoses, treatment options, outcome measures, as well as quality of life studies are discussed.

## Introduction

In 1972, Gideon et al. first described an inflammatory bone disease of subacute or chronic nature that affects bones with a symmetrical and multifocal pattern [1]. Since then, a multitude of descriptive terms has been used throughout different medical subspecialties (orthopaedics, infectious diseases, paediatric and adult rheumatology) to describe the disease. From a current standpoint, chronic non-bacterial osteomyelitis (CNO) seems to have become the consistent denomination describing chronic bone inflammation independent of its distribution (unifocal vs. multifocal) or course (self-limited, chronically active vs. recurrent) [2]. Subsequent considerations in the 1980s identified chronic recurrent multifocal osteomyelitis (CRMO) as a severe form of CNO [3, 4].

In the past, a significant fraction of the patients received antibiotic therapy in the initial treatment, when the diagnosis was unclear, but also in part based on the reported occasional isolation of *Propionibacterium acnes*, currently named “*Cutibacterium acnes*”. However, starting in the 1990s, the infectious nature of the disease was questioned, because of increasing reports on sterile lesional biopsies and failure to detect bacterial pathogens using molecular techniques, including PCR [5].

More recently, the molecular basis of chronic inflammation has been linked to a pro-inflammatory phenotype of monocytes from CNO patients with increased expression of inflammatory interleukin (IL)-1β, IL-6 and TNF-α [6, 7], and reduced expression of immune regulatory IL-10 and IL-19 [8, 9••]. The resulting dysbalance between pro- and anti-inflammatory pathways contributes to chronic tissue inflammation, osteoclast activation, bone destruction, and in some cases hyperostosis and sclerosis of bone. In addition, mast cells have been identified to contribute to sterile inflammation [10]. The molecular basis of CNO has also been analysed in mouse models of chronic bone inflammation and in patients with familial forms of CNO or (monogenic) autoinflammatory bone disease with bone involvement. Significant knowledge has been gained by the identification of gene mutations associated with bone inflammation in mice (*Pstpip2*) and humans (*IL1RN*), predominantly leading to NLRP3 inflammasome activation, the production of pro-inflammatory cytokines IL-1β, TNF-α and IL-18, and/or their reduced regulation [9••, 11, 12••].

Several authors have linked CNO to the concept of spondyloarthropathies (SPA) [13]. However, in most cohorts, HLA-B27 positivity ranges around frequencies in the normal healthy population or is mildly elevated, and male predominance is not present [14, 15••]. Further classical features of SPA, such as uveitis or urethritis, are also not present [15••]. Clinical and pathophysiologic proximity of CNO to the so-called SAPHO syndrome, that usually affects adults, and overlap with psoriasis syndromes have also been considered. The acronym SAPHO stands for synovitis, acne, pustulosis, hyperostosis and osteitis syndrome and was first described in 1987. Since then, a considerable overlap between both entities, SAPHO and CNO/CRMO, has been discussed, which is mostly based on inflammatory cutaneous manifestations in a subset of CNO patients [16–18, 19••, 20]. Furthermore, also in adult SAPHO syndrome patients, features of spondyloarthropathies are present. Of note, the possible connection to SPA dominates the SAPHO literature much more than in paediatric CNO [21].

Following the historic definition of systemic or organ specific inflammation in the absence of self-reactive lymphocytes and high-titre autoantibodies, and the aforementioned central involvement of innate immune mechanisms, CNO and SAPHO have been classified as autoinflammatory diseases [22, 23].

## Clinical Presentation and Laboratory Findings

Inflammatory bone lesions may be uni- or multifocal. With the exception of the neurocranium, which is almost never affected in CNO, inflammation can affect all sites of the skeleton [2]. Typical sites of inflammation include the metaphyses/and epiphyses [24] of long bones of the extremities, the shoulder girdle including the clavicle and the sternum, vertebral bodies, and (in rare cases) the mandible. From a patients’ view, recurrent clinical symptoms, including local swelling, pain and impairment of motion, are common complaints. Imaging techniques suggest a primary chronic course of periosteal and/or endosteal inflammation, osteitis and osteomyelitis. Clinically and also radiologically, arthritis of adjacent or remote joints may be present. In a few cohorts, a strong clinical link towards SPA has been noted [13]. However, this does not seem to be a general feature in children and adolescents [15••].

Other organ systems may be involved as well. Up to 20% of CNO patients develop skin manifestations, including palmoplantar pustulosis, cystic acne and psoriasis [15••]. Chronic inflammatory bowel disease may be present in up to 10% of patients [25, 26]. Occasionally, hepatosplenomegaly and lymph node enlargement are noted (up to 3% of patients). Ocular or cardiac manifestations are generally rare. Uveitis or episcleritis are no typical features of CNO [15••].

Laboratory tests usually show mild to moderate elevation of inflammatory parameters, including CRP and ESR. Preliminary reports promise potential for a set of serum proteins to act as biomarkers for CNO. These include the pro-inflammatory cytokine IL-6, the chemokine CCL-11/eotaxin and others, and may help to differentiate CNO patients from JIA and osteoarticular infections [27]. However, findings need to be validated using alternative technologies in unrelated multi-ethnic cohorts. Thus, and due to the limited general availability, the clinical relevance of these findings is currently limited. Based on register data from Europe, antinuclear antibodies (ANA) are present in up to 39% of patients [15••]. However, the proportion of ANA-positive CNO patients in large individual cohorts is usually much lower, ranging around 10%. The number of lesions, the clinical picture, inflammatory laboratory parameters and the response to treatment did not differ between ANA-positive and ANA-negative patients [15••]. Thus, the relevance of ANA positivity in CNO requires to be challenged and elucidated further. For now, no conclusive clinical or laboratory autoimmune features have been reported.

Taken together, in the absence of reliable and widely accepted laboratory tests, disease biomarkers or known genetic causes, the definition and classification of CNO have to rely on descriptive parameters. Several attempts have been made to develop classification or diagnostic scores for CNO and to separate its clinical picture/symptoms from acute bacterial osteomyelitis or malignant bone disease. All include CNO typical patterns of bone inflammation or monofocal bone disease with histological features resembling chronic inflammation in the absence of malignancy, the absence of other signs severe systemic inflammation (such as fever, high CRP and/or ESR), and chronic disease courses. As such, scores stay descriptive and have not been validated prospectively and/or in unrelated cohorts [28–30].

## The Role of Histopathology and Microbiology

In patients who do not exhibit clinical symptoms and/or radiographic findings conclusive for CNO, a biopsy of the/a representative lesion is necessary. Tissue sections should be read by pathologists experienced with bone malignancies and CNO. First and foremost, malignancy (primary bone tumours, metastases, lymphoma, etc.) and systemic disease (Langerhans cell histiocytosis) require to be excluded. While some features, such as coexistence of “acute” inflammatory infiltrates (neutrophils, macrophages) with chronic inflammation (characterised by lymphocytes, plasma cells and monocytes), and/or bone sclerosis, are more common in CNO than other conditions, they are not specific. Chronic infections e.g. may deliver similar histopathological patterns [5, 31, 32].

Thus, fresh (not paraffin embedded) bone tissue requires to be tested for infectious agents. Microbial analyses should include long-term standard cultures, including the search for mycobacteria. In addition, molecular microbial analysis of biopsies, including universal microbial rRNA amplification, mycobacteria search PCRs and possibly next-generation sequencing (NGS, where available and of interest), may be performed. In most patients, non-suspicious microbial results will be noted.

However, in individual cases, *Propionibacterium/Cutibacterium acnes* may be present, which complicates the diagnosis as they may represent contamination [5, 33]. Thus, a clinical dilemma exists as acute or chronic bacterial osteomyelitis may not exhibit high inflammatory parameters, especially when low-virulent strains of bacteria are present, such as *Propionibacteriae* [34, 35].

The better the individual physician or clinical division is acquainted with diagnosing CNO, the fewer biopsies will be performed. During clinical work-up until infectious osteomyelitis is excluded, an initial antibiotic therapy may be reasonable. However, if the clinical symptoms resemble those “typical” for CNO, antibiotic therapy may be omitted. Of note, throughout international cohorts, antibiotic therapy has been reported in as many as 38% of patients [15••].

In SAPHO, as in CNO, the fundamental clinical component is inflammatory osteitis, which may result in hyperostosis. Most frequently affected regions include the rib cage (ribs and sternum), the spine and long bones of the extremities. This largely resembles the pattern in CNO [36]. Since arthritis/synovitis and acne are included in the acronym, it appears that SAPHO is closely related to childhood CNO, but covers bone inflammation in the context of associated cutaneous manifestations in a single individual. This association is certainly present, but less common in classical paediatric CNO. However, since in the overall adult population beyond SAPHO patients, acne and pustulotic skin lesions are more prevalent as compared with children and young adolescents, a confounding factor may be present. Of note, one study reported that up to 67% of bone biopsies from adult SAPHO patients were positive for *Propionibacterium acnes* [37]. In this context, it is interesting to note that *Propionibacterium acnes* can trigger increased plasma levels of the chemokine IL-8 and pro-inflammatory cytokines IL-18 and TNF-α. This may be caused by stimulation of the Toll-like receptors (TLR) 2 and 4 by *Propionibacterium acnes* [38, 39]. However, primary antibiotic therapy for SAPHO syndrome seems only effective as long as it is administered [37]. This led to the conclusion that the presence of this bacterium at the site of the lesion or in the skin might not be the only causative trigger of the disease, but of relevance as it may amplify inflammation in otherwise predisposed individuals. In addition, observed effects of antimicrobials may also partially be explained by anti-inflammatory effect of the drug studied (azithromycin). With regard to HLA-B27, no consistent presence above the expected regional frequencies was noted also in SAPHO [40]. In this respect, SAPHO mimics CNO. Though SAPHO syndrome is usually described in late adolescents and adults; some cases of paediatric manifestations have been reported [41]. Studies including both children and adults are rare. Where comparisons are possible, adult patients sometimes may have more skin involvement, but show a comparable distribution of bone lesions. Lastly, treatment available appears less effective in adults as compared with children [42•, 15••].

## Molecular Pathophysiology in Humans and Mice

The molecular pathophysiology of “sporadic” CNO/CRMO (not following Mendelian inheritance) is incompletely understood. There is a significant need to analyse pathophysiological pathways, since not only inflammatory components but also potentially post-infectious reactive features have been observed.

Monocytes isolated from peripheral blood of CNO/CRMO patients fail to produce the immune regulatory cytokine IL-10 (and its homologue IL-19) in response to stimulation with lipopolysaccharide (LPS). This has been linked with reduced activation of mitogen-activated protein kinases (MAPK) ERK1 and 2, which results in reduced activation of the transcription factor signalling protein (SP-)1 and reduced phosphorylation of histone H3 at serine position 10 (H3S10P). Reduced H3S10P results in epigenetic “closure” of the *IL10* promoter, which, in context with reduced availability of SP-1, translates to reduced IL-10 expression. At the same time, pro-inflammatory IL-6, IL-20 and TNF-α are expressed at increased levels. This may be secondary to increased activation of the NLRP3 as absence of IL-10 primes inflammasome assembly and subsequently may lead to release of IL-1β, which triggers TNF-α and IL-6 expression [7, 20, 43]. Of note, in monocytes from CNO patients, DNA methylation of inflammasome associated genes *IL1*, *PYCARD* (encoding for the adaptor molecule ASC) and *NLRP3* is reduced, resulting in increased gene expression [32]. However, the exact chain of events causing the significant cytokine imbalance in CNO has yet to be determined. In line with these observations, tissue analyses of bone biopsies revealed a significant deregulation/activation of the IL-1β axis [6, 9••]. Finally, these altered cytokine expression patterns may lead to increased activation of osteoclasts via the RANK/RANK ligand signalling pathways, directly affecting bone remodelling [8]. Interestingly, comparable pathophysiological findings have been seen in the serum of SAPHO patients [44].

Monogenetic systemic autoinflammatory disorders with bone involvement partially resemble “sporadic” CNO, demonstrating bone inflammation, osteolysis and hyperostosis. CRMO, the severe form of CNO, shares clinical features with chronic auto-inflammatory diseases, including deficiency of the IL-1 receptor antagonist (DIRA), and pyogenic arthritis, pyoderma gangrenosum and acne (PAPA) syndrome. Both syndromes may show lesions of cartilage and osteolysis [19]. In Majeed syndrome [45], an autosomal recessive inheritance has been documented with mutations in the lipin-2 gene (*LPIN2)* [46]. In addition to multifocal bone lesions resembling CNO, dyserythropoietic anaemia and pustulous dermatosis may be present.

Monogenic dominant mutations in the SH3 domain binding protein 2 (*SH3BP2*) can lead to a granulomatous bone lesion of the lower jaw, named hereditary cherubism, partially resembling paediatric CNO of the mandibles [19, 47].

Recently, Cox et al. reported homozygous mutations in the filamin-binding domain of *FBLIM1* as well as other types of mutations in about 1% of their cohort patients, leading to bone inflammation [48]. Of note, no other paediatric cohort has been analysed genetically in a comparable detail. *FBLIM1* analysis has been performed in an adult SAPHO patient cohort, but did not provide evidence of relevance in a German cohort (Assmann G et al., 2020 submitted, in press, permission given in personal communication).

A point mutation in the *FGR* gene, encoding for the Gardner-Rasheed feline sarcoma protein kinase, a member of SFK family (src family tyrosine kinase), has been demonstrated in Ali18 mice that resemble a model for sterile osteomyelitis. In a large cohort, approximately 13% of CNO patients also carried rare exonic variants in this particular *SFK* gene [49].

Further monogenic diseases resemble CNO in part: Bone inflammation has been described in familial hyperphosphatemic familial tumoral calcinosis affecting the *DALNT3* gene [50], in addition to primary hypertrophic osteoarthropathy, revealing gene mutations in the prostaglandine metabolism and hypophosphatasia with mutations in the tissue non-specific alkaline phosphatase gene (reviewed in [19]).

Besides the already mentioned Ali18 mice, further mouse models affecting the proline-serine-threonine phosphatase interacting protein 2 (*Pstpip2*) gene have been described. The phenotype of these mice shows sterile inflammation of bone, cartilage and skin. Inflammation is mediated by chemokines and cytokines, including M-CSF, MIP-1α and IL-1β. Particularly the *Pstpip2* gene is tempting as a potential target for CNO to be analysed, since on the other side mutations in the *PSTPIP1* gene are implicated in the pathogenesis of another autoinflammatory syndrome, the PAPA syndrome. However, no conclusive mutations in *PSTPIP1* or *2* have been revealed in sporadic CNO patient cohorts [51–53].

**Fig. 1:**
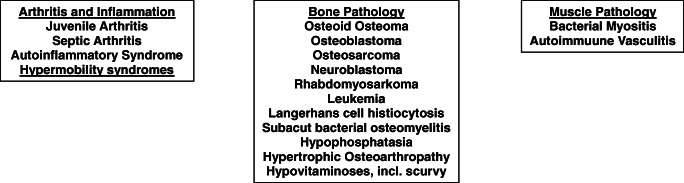
Differential diagnosis of chronic non-bacterial osteomyelitis in childhood

Though only few patients with suspected “sporadic” CNO cohorts may indeed be affected by rare mutations in single genes, keeping these differential diagnoses in mind is of relevance, especially in very young children with CNO. Target-directed treatments aiming for the correction of the biochemical sequels may already be available for rare conditions, e.g. enzyme replacement therapy in hypophosphatasia or recombinant IL-1 receptor antagonist replacement in DIRA. For a (potentially incomplete) summary of relevant differential diagnoses, see Fig. 1.

## Imaging Strategies

The diagnoses of exclusion CNO and SAPHO rely predominantly on imaging techniques. A number of differential diagnoses remains to be considered as long as generally accepted and validated diagnostic criteria including genetics and/or biomarkers are missing. Over the past years, an international expert group has been working towards the identification of CNO-related symptoms and their classification, including imaging findings. This exercise aims at the harmonization of diagnostic approaches and treatment in CNO [54, 55].

Diagnostic imaging frequently includes conventional X-rays and/or regional magnetic resonance imaging (MRI) at sites of pain. Occasionally, computed tomography (CT) is used if MRI is unavailable or does not deliver sufficient detail in depicting the bony structure. In children, CT imaging should be avoided. Whole body imaging is a central imaging tool for the diagnosis of CNO and is performed to monitor for additional sites of bone inflammation, especially in the vertebral column. Whole body (WB-)MRI is the gold standard to screen for additional, potentially clinically silent lesions. In the past, ^99m^technetium bone scintigraphy was used, but should be considered obsolete and only be used if whole body MRI/MRI is not available [24, 56].

Initially during clinical care, X-ray images are frequently taken to exclude differential diagnoses e.g. bone fractures and may (but certainly do not have to) show CNO-associated changes. Conventional X-rays may already show osteolytic lesions or hyperostotic features. Especially long bones of the extremities are prone to such changes, which appear later in the course of disease. Fractures of long bones are very rare. However, fractures can occur in vertebrae of up to 10% of CNO patients [57]. Conventional X-rays may also be normal and usually cannot distinguish CNO from the features of malignant bone diseases, such as osteosarcoma or Ewing’s sarcoma.

**Fig. 2:**
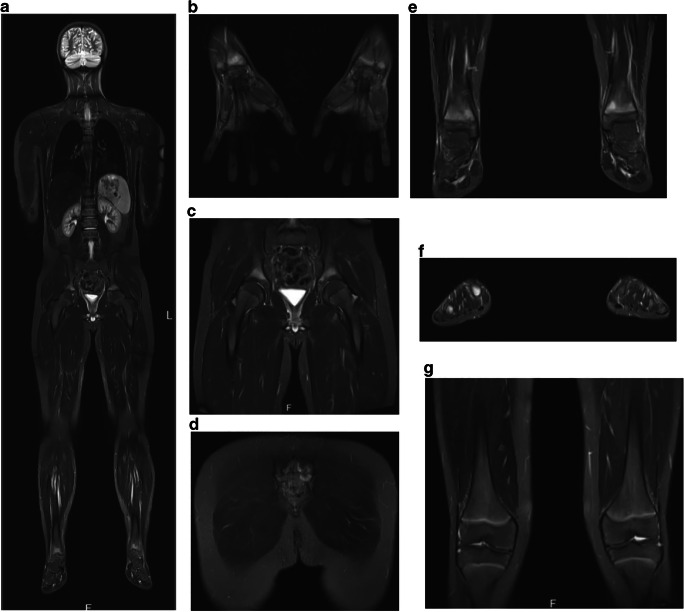
Whole body MRI of CNO. Whole body MRI using turbo inversion recovery measurement (TIRM) (**a**) of a 12-year-old male patient exhibiting symmetrical inflammatory bone lesions of the distal radius (**b**), distal tibiae (**e**), iliac bones (**c**), the sacral bone (**d**) and metatarsal bones (**f**). Knees were not affected (**g**)

Currently, MRI studies are the imaging gold standard in CNO. Usually, at the time of diagnosis, regional MRIs are performed and can help to diagnose CNO and exclude differential diagnosis [58]. In the authors’ institutions, regional MRIs using TIRM (or STIR) sequences are used to localize and assess the extent of inflammation, followed by native T1 sequences and (not always) contrast-enhanced T1-weighted sequences with fat saturation to assess regional changes and surrounding tissue involvement. At diagnosis, WB-MRI provides information on the distribution of bone lesions, soft tissue involvement and/or possible additional organ involvement which may be present in malignant disease (such as neuroblastoma). Noteworthy, based on growing experience, the use of MRI contrast media can be omitted during follow-up investigations or if initial WB-MRIs at diagnosis show CNO typical multifocal patterns [59] (Fig. 2). Especially during long-term follow-up, repetitive (WB or regional) MRI can be used for assessing the extent and distribution of lesions and, thus, severity of CNO in order to guide treatment.

Though it should not be used for the differential diagnosis of CNO in children, because of radiation and general superiority of MRI, CT may be helpful in rare occasions to exclude differential diagnoses (e.g. detection of the nidus in osteoid osteoma or osteoblastoma) [60]. Based on reports in the literature, for diagnosing and management of SAPHO syndrome in adults, CT appears to be of higher diagnostic relevance in clinical practise when compared with paediatric CNO [39, 42•].

## Treatment, Monitoring and Treat-to-Target Protocols

### CNO Treatment

Currently, no treatments are approved for the use in CNO. However, there is general consensus that CNO patients benefit from anti-inflammatory therapies using non-steroidal anti-inflammatory drugs. If NSAIDS are administered on a regular and controlled basis, a significant portion of patients (up to 70%) may reach a symptom-free state after 18 months [2]. Based on the current pathophysiological understanding of CNO, NSAIDs may (at least partially) correct increased osteoclast activity through the reduction of prostaglandin production (inhibition of cyclooxygenase enzymes). Prostaglandins are essential for osteoclast differentiation and activation. Furthermore, NSAIDs affect pain processing which explains their more immediate analgesic effects [8]. In order to estimate their overall effectiveness, a recent long-term retrospective study demonstrated that 50% of NSAID-treated CNO patients may develop flares after a median of 29 months [61]. In cases refractory to NSAID treatment, patients may benefit from short courses of corticosteroid therapy, in addition to NSAIDs.

Defining the therapeutic target together with patients and families is of particular importance in CNO. In light of this, even in patients in “full clinical remission”, MRI may still document altered and hyperintense bone signals (“active bone lesions”). To address this, a disease activity score (ped CNO-score) has been developed that includes the number of bone lesions, in addition to inflammatory lab parameters [62]. As long as no structural damage, especially to the spine, is noted and the patient is improving by clinical means, non-steroidal anti-inflammatory medication may serve as the only medication used, especially if the “target” is a pain-free condition. In patients with a multifocal pattern and/or vertebral structural involvement, or patients experiencing relapses or insufficient response to NSAID, the “target” should focus not only pain control and improvement of impairments; the “target” considerations should also include a lesion-free MRI.

Classical disease-modifying antirheumatic drugs (DMARDs), such as sulfasalazine or methotrexate (MTX), may be used to reach this target as well as cytokine blocking strategies/biologic DMARDs, usually TNF-α blocking medications, or bisphosphonates [2, 8, 61, 63]. Based on the abovementioned pathophysiological models, corticosteroids may (similar to NSAIDs, but through inhibition of phospholipase A) inhibit osteoclast differentiation and activation. Furthermore, corticosteroids reduce the expression of NFkB-dependent pro-inflammatory cytokine expression while increasing anti-inflammatory cytokine expression (including IL-10) [8]. In the authors’ institutions, corticosteroids are usually used in short “oral bursts” of 5–10 days to induce rapid control of bone inflammation. Some authors use corticosteroids for several weeks to induce remission and cover until classical DMARDs are developing full efficacy [54]. Cytokine blocking strategies in CNO usually consist of TNF inhibitors, which (at least partially) correct the imbalance in pro- vs. anti-inflammatory cytokine expression [61] and appear effective in patients who failed to respond to the aforementioned treatment options. Vertebral involvement without structural damage may be subjected to TNF inhibition. Based on the current pathophysiological understanding, IL-6 or IL-1 blocking agents also promise therapeutic potential. Individual cases reported in the literature suggest efficacy of treatment with recombinant IL-1 receptor antagonist (anakinra) in some cases [64].

In cases of spinal bone affection/destruction and/or severe multifocal CNO, long-term sequelae including scoliosis may result. Thus, bisphosphonates have been suggested in this type of manifestation and documented to be a good treatment option in several cohorts [4] [15••, 54, 65–67]. Furthermore, bisphosphonates are an alternative to biologic DMARDs in otherwise treatment refractory cases or individuals with involvement of the vertebral spine or mandible. Some authors claim that they should be used first line for vertebral involvement with structural damage (fractures) [8, 54, 61]. Bisphosphonates inhibit osteoclast activity and may (partially) correct the imbalance between pro- and anti-inflammatory cytokines in a yet to be determined manner (reviewed in [8]).

**Fig. 3:**
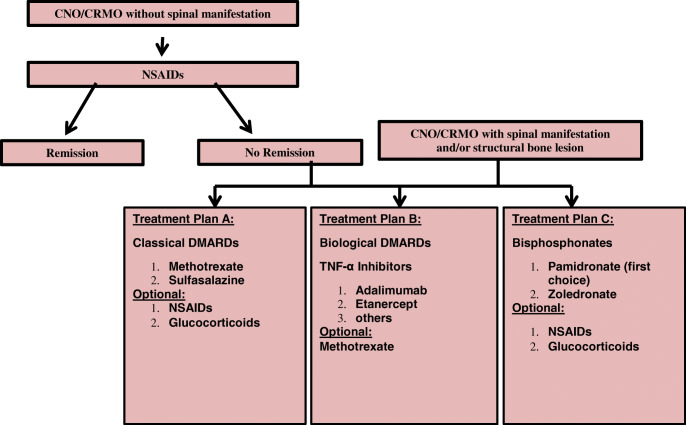
Proposed treat-to-target protocol for childhood CNO

Because, with the exception of one controlled prospective follow-up study using NSAID, no prospectively collected data exists investigating treatment responses in CNO [62], an international initiative (led by the North American CARRA group) provided consensus diagnostic and treatment plans for CNO patients who fail to respond to NSAID treatment or have vertebral involvement [54]. The “target” of therapeutic strategies should be based on the consensus with the patients and parents. Ideally, the goal may be a symptom-free state in addition to the reduction of signs of inflammation or remission on WB-MRI imaging. However, this latter goal usually comes along with a necessary long-term treatment approach, especially if MRI changes are still present, but the patient may not experience complaints any more. Acceptance of further therapy might be limited in this constellation [54, 62]. The mentioned international initiative aims at homogenised diagnostic approaches and treatment protocols, and will prospectively collect treatment response data [54] (Fig. 3).

### SAPHO Treatment

Evidence-based data on SAPHO syndrome are in urgent need as randomized controlled trials also do not exist. Non-steroidal drugs are generally used as first-line treatment with limited effectiveness as compared with paediatric CNO. Taking into account the potential disease amplifying role of bacteria, e.g. *Cutibacterium acnes*, some authors suggest beneficial effects in the administration of antibiotics (such as azithromycin, doxycycline, clindamycin and sulfamethoxazole/trimethoprim) [37, 68, 69]. Systemic or intra-articular corticosteroids may have positive effects, but their use is limited due to side effects and limited long-term efficacy [70, 71]. Bisphosphonates are used regularly, especially if the spine is affected [40, 72]. A not randomized study recently showed significant improvement of symptoms in response to treatment with pamidronate for spinal bone marrow edema, leading to the authors’ recommendation to use pamidronate as first-line treatment especially in cases with spinal involvement [73].

Classical DMARDs, such as methotrexate or sulfasalazine, are widely used in SAPHO with inconclusive results [40]. In otherwise treatment refractory cases, biologic DMARDs can be introduced [74]. Several biological DMARDs have been described with beneficial outcome [40]. In addition to anti-TNF agents that have positive effects on musculoskeletal manifestations and partly on the skin, other cytokine-blockers are used. Daoussis et al systematically reviewed published case studies and case reports focussing on biologic DMARD treatment [75]. Preliminary reports suggest IL-1 blockade with anakinra to have beneficial effects in osteitis and arthritis [76], but variable effects on mucocutaneous involvement [77]. Due to its successful use in psoriasis treatment, secukinumab, an IL-17A neutralizing antibody, was used in SAPHO patients mainly affected by skin manifestations, but also positive effect on osteitis was seen. Thus, the use of IL-17-blockade may be another therapeutic option in otherwise refractory cases [75]. Individual case reports exist on successful treatment of SAPHO with apremilast, a PDE4-inhibitor [78], or the JAK inhibitor tofacitinib [79, 80].

### Disease Outcomes and Quality-of-Life

Long-term experience strongly suggests that using anti-inflammatory medication “on demand” may not improve the long-term outcome of CNO. Data on controlled treatment duration is limited: Patients with multifocal disease have been reported to reach remission after 1.5 years [2]. However, up to 50% of patients may relapse after approximately 2.5 years, demonstrating the chronic nature of bone inflammation in CNO [61]. Generally, the prognosis of CNO is considered reasonably good, if an anti-inflammatory medication is consequently administered [61, 62]. In case of an insufficient treatment response to NSAIDs or with vertebral involvement, conventional or biological disease-modifying agents or bisphosphonates should be considered. In the absence of licensed treatment options and/or randomised controlled trials, treatment should be planned following the aforementioned international consensus protocols [54]. Though patients experience significant pain, negatively affecting their quality-of-live and/or psychosocial development, long-term quality-of-life and disease outcomes in CNO patients have been reported to be good when treated appropriately [81].

## Conclusions

CNO/CRMO and SAPHO are autoinflammatory conditions with a strong involvement of cytokine dysregulation. While paediatric CNO is primarily associated with increased activation of innate immune mechanisms and responds to blockade of these, SAPHO patients respond to blockade of IL-17A, a primarily T lymphocyte-derived cytokine. This may support variable pathomechanisms in adult SAPHO patients as compared with paediatric CNO with adaptive immune cells playing a more pronounced role in SAPHO syndrome. While this is not scientifically proven, T cell involvement in SAPHO may reflect secondary activation of adaptive immune mechanisms as (at least some) SAPHO patients may have experienced “isolated” CNO before the onset of skin symptoms. Prospective collection of treatment response data alongside biosamples for molecular studies may result in patient stratification and individualised treatments. This requires national and international collaborations such as initiated by the PRES Eurofever initiative [15••] or the North American CARRA group in association with many international partners [54].

## Abbreviations

ANA: Antinuclear antibodies
CNO: Chronic non-bacterial osteomyelitis
CRMO: Chronic recurrent multifocal osteomyelitis
CT: Computed tomography
DIRA: Deficiency of interleukin-1 receptor antagonist
DMARD: Disease-modifying anti-rheumatic drug
ERK: Extracellular signal-regulated kinase
FBLIM: Filamin-binding LIM protein
G/M-CSF: Granulocyte and monocyte colony stimulating factor
HLA: Human leucocyte antigen
IL: Interleukin
IL1 RN: Interleukin-1 receptor antagonist
LPIN2: Lipin-2, a phosphatidate phosphatase
MAP kinase: Mitogen-activated protein kinase
MCP-1: Monocyte chemotactic protein 1 (also CCL2)
MIP: Macrophage inflammatory protein
MRI: Magnetic resonance imaging
MTX: Methotrexate
NGS: Next-generation sequencing
NLRP: NACHT, LRR and PYD domains-containing protein
NOMID: Neonatal onset multisystemic inflammatory syndrome
NSAIDS: Non-steroidal anti-inflammatory drugs
PAPA: Pyoderma gangrenosum and acne syndrome
PBMC: Peripheral blood mononuclear cells
PCR: Polymerase chain reaction
PSTPIP: Proline-serine-threonine phosphatase-interacting protein
RANK: Receptor activator of nuclear factor-κB
RANKL: Receptor activator of nuclear factor-κB ligand
SAPHO: Synovitis acne pustulosis hyperostosis osteitis syndrome
SPA: Spondyloarthropathy
SFK: src family tyrosine kinase family
TIRM: Turbo inversion recovery measurement
TLR: Toll-like receptor
TNF: Tumour necrosis factor
TRAPS: TNF receptor associated periodic syndrome
